# Learning about the Functions of the Olfactory System from People without a Sense of Smell

**DOI:** 10.1371/journal.pone.0033365

**Published:** 2012-03-21

**Authors:** Ilona Croy, Simona Negoias, Lenka Novakova, Basile N. Landis, Thomas Hummel

**Affiliations:** 1 Smell and Taste Clinic, Department of Otorhinolaryngology, University of Dresden Medical School, Dresden, Germany; 2 Department of Psychosomatic Medicine, University of Dresden Medical School, Dresden, Germany; Alexander Flemming Biomedical Sciences Research Center, Greece

## Abstract

The olfactory system provides numerous functions to humans, influencing ingestive behavior, awareness of environmental hazards and social communication. Approximately ⅕ of the general population exhibit an impaired sense of smell. However, in contrast to the many affected, only few patients complain of their impairment. So how important is it for humans to have an intact sense of smell? Or is it even dispensable, at least in the Western world? To investigate this, we compared 32 patients, who were born without a sense of smell (isolated congenital anosmia - ICA) with 36 age-matched controls. A broad questionnaire was used, containing domains relevant to olfaction in daily life, along with a questionnaire about social relationships and the BDI-questionnaire. ICA-patients differed only slightly from controls in functions of daily life related to olfaction. These differences included enhanced social insecurity, increased risk for depressive symptoms and increased risk for household accidents. In these domains the sense of olfaction seems to play a key role.

## Introduction

The olfactory system provides many functions for humans, influencing ingestive behavior, increasing awareness of environmental hazards and social communication (for overview see [1]). For example, the olfactory system is important for detecting food and providing good taste quality, for avoiding potential dangerous situations in long- and short distance, like fire and microbial threats. Additionally olfaction seems to play a key role in mate choice and helps to detect emotions in other people [2].

But does olfaction enrich information from other sensory systems, like the visual, tactile or auditory senses? Does it thus allow us to experience the world more deeply? Or does the olfactory sense possess functions of it own, which cannot be fulfilled by other systems? A number of extensive studies from various countries indicate that approximately 15–20% of the population exhibits some olfactory loss, and that 2.5–5% exhibit functional anosmia. However, one more recent study [3] indicated that 3.8% of the population exhibit severe olfactory loss. These differences in numbers seem to relate to differences in the interpretation of test results and differences in the study design [3], [4], [5], [6], [7], [8]. Despite these differences, it appears that a relatively large portion of the general population exhibits olfactory loss. Unlike eyeglasses for visually impaired people, there is no compensation for an impaired sense of smell. But relative to the large portion of people who are affected, only few complain. So how important is it for humans to have an intact sense of smell? Is it even dispensable, at least in the Western world?

One approach to answer this question is to ask people with acquired olfactory disorders what they miss. Those patients typically complain about difficulties with cooking, a lack of appetite and low interest in eating [9], [10]. In addition, they are subject to an increased risk for hazardous events [11], [12]. Furthermore these patients report daily life problems associated with social situations [13] and concerns about their body odor [14]. About 17 to 30% of patients with olfactory disorders report decreased quality of life including symptoms of depression [14], [15], [16]. The loss of quality of life is most severely perceived by younger patients with poor olfaction [17], [18] (for overview see [19]).

But studying people with an acquired olfactory disorder might lead to distorted results: it may be difficult to determine if their problems result from the loss of olfactory ability, rather than its absence.

Therefore, we aimed to study people who were born without a sense of smell. Among clinicians, this phenomenon is known as **Isolated congenital anosmia (ICA)**. ICA is characterized by the lack of the sense of smell since birth in otherwise healthy people [19]. In the Smell and Taste Clinic at the Department of Otorhinolaryngology of the University Medical School Dresden we see approximately 20 patients per year diagnosed with ICA. Diagnosis of ICA involves a detailed medical history and psychophysical examination, electrophysiological measurements and magnetic resonance imaging with special focus on the structure of the olfactory bulb [20]. Hypoplastic or aplastic olfactory bulbs in otherwise healthy participants are typically found, accompanied by a shallow olfactory sulcus [20]. Based on our data, we estimate the prevalence of ICA in the general population to be 1: 5000–10000.

Kallmann's syndrome is an important differential diagnosis characterized by the lack of the olfactory bulb and hypogonadotropic hyopogonadism. The incidence of this disorder is estimated at about 1∶86.000 in the general population [21]. Because the hormonal dysfunction in Kallmann's syndrome causes a lot of additional problems, those patients are explicitly not included in the study.

Although there are very few studies and single case reports about patients with ICA, based on the literature and on our experience, we can report that they typically do not complain about a reduced quality of life [9]. The patients are mostly unaware of the olfactory deficit as children [22] and rather the parents become suspicious that something might be “wrong”. Typically this occurs when it is obvious that the child is not disturbed by bad smells, like rotten milk, dog's feces, or smells during chemistry lessons at school. However, to our knowledge, there is no systematically collected evidence on the lifestyle of people born without a sense of smell. If the sense of smell is important for ingestive behaviour, environmental hazards and social communication, like described above, how are these domains affected in patients with ICA? Do ICA patients have trouble maintaining their weight or do they obtain no joy in eating, for example? Do they accidentally eat spoiled food? Do they also worry about their body odor? And do they feel different in social situations? Or are people without a sense of smell not affected at all by this deficit and is olfaction just overestimated?

In this study, we wanted to provide a very first step in answering those questions. In a hypothesis-generating design we sent a broad questionnaire covering these topics to patients with ICA.

## Methods

### Ethics Statement

The study followed the Declaration of Helsinki on Biomedical Research Involving Human Subjects and was approved by the Ethics Committee from the University of Dresden Medical School. All participants provided written informed consent.

### Participants

Originally 50 ***patients***, diagnosed with ICA, participated in the study. Because daily life functioning depends on age, we decided to exclude participants older than 60 and younger than 18 years to get a more focused age group. Therefore the questionnaires of 32 participants (aged 18–46years, mean age 31+/−8 years) were analyzed for the study. Congenital anosmia was diagnosed using detailed medical history, psychophysical examination, electrophysiological measurements and magnetic resonance imaging.

**Figure 1:**
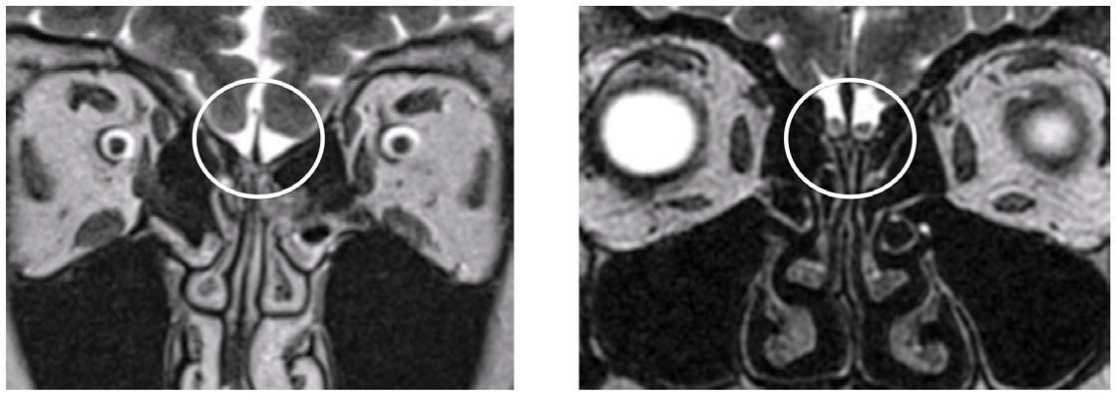
Structural magnetic resonance image of an isolated congenital anosmic patient (left). Within the marked region an olfactory bulb is missing. This becomes obvious compared to the healthy person visualized in the right picture.

Psychophysical examination was performed using the Sniffin' Sticks, including tests for olfactory threshold, discrimiation and identification ability (“Sniffin' Sticks”, Burghart GmbH, Wedel, Germany; compare Hummel, 2007). Additionally patients underwent electrophysical measurement. Trigeminal (CO_2_) and olfactory (PEA, H_2_S) stimuli were presented to patients in order to record event related potentials. Chemosensory nasal stimulation was performed using a stimulator (Olfactometer OM2S, Burghart Instruments, Wedel, Germany), which allows administration of chemical stimuli without causing concomitant mechanical or thermal sensations. Anosmic patients do not show event related potentials in response to olfactory stimuli, but they typically do exhibit response to trigeminal ones. Additionally patients underwent structural magnetic resonance imaging (compare Yousem et al 1996). If no olfactory bulb could be found by a trained physician, if no hint of olfactory function could be found in any of the tests performed, if patients had no memory of ever having been able to smell something and other possible causes of congenital anosmia were excluded (e.g. Kallman's syndrome), isolated congenital anosmia was diagnosed. For illustration, we show a structural magnetic resonance image of an ICA patient in Figure 1 in comparison to an image obtained in a healthy control subject.

Thirty-six age-matched healthy participants (aged 18–50 years, mean age 29+/−7 years; Table 1) served as control group. They were recruited from our database of healthy participants, that took part in other studies. The actual status of health was checked by detailed medical history, olfactory function was checked by an olfactory screening test [23]. Normosmic function was ascertained in all controls. Patients and controls did not differ significantly with regard to age and sex distribution.

**Table 1:** Descriptive statistics and comparison between ICA-patients and controls.

			ICA-patients (N = 32)			Healthy controls (N = 36)			Group comparison
			mean	SD	N	mean	SD	N	p-value
	Age		30.50	7.65		29.33	6.57		n.s.*
	Sex	Female			22			21	n.s.+
		Male			10			15	
**Ingestion**	Size in cm		170.75	9.52		173.60	9.24		n.s.*
	Weight in kg		69.73	12.60		69.94	14.22		n.s.*
	Body mass index		23.81	3.25		23.10	3.71		n.s.*
	Breast-fed	No			7			6	n.s.+
		Yes			25			29	
	Components of preferred food		1.43	.69		1.23	.43		n.s.*
	Eating behavior		2.21	.54		2.26	.47		n.s.#
**Environ-mental hazards**	household accidents		2.02	.52		1.51	.42		<0.001#
	Washing behavior		2.25	.65		2.16	.66		n.s.#
	Frequency of showering	More than daily			0			1	n.s.#
		Daily			16			24	
		Every two days			10			8	
		More than weekly			4			3	
		Weekly			1			0	
		Rarely			1			0	
**Social behavior and communication**	Partnership status	Married			8			1	n.s.+
		Divorced			2			2	
		Single			10			17	
		Engaged			12			16	
		widowed			0			0	
	Satisfaction with partnership								n.s.#
	Number of children		.53	.80		.33	.79		n.s.*
	Age of first sexual intercourse		18.10	2.65		19.00	4.76		n.s.*
	Sexual satisfaction		1.76	0.76		1.73	0.81		n.s.#
	Number of sexual relationships		3.34	3.89		6.2	7.00		0.048*
	BBE-Questionnaire	Security mother	4.06	.90		4.38	.62		n.s.*
		Security partner	4.31	.63		4.64	.36		n.s.*
		Dependency mother	2.01	.63		1.96	.60		n.s.*
		Dependency partner	2.85	.61		2.82	.45		n.s.*
	social worries		2.18	.51		1.49	.36		<0.001#
**Depression**	BDI-Questionnaire		10.47	9.38		4.63	6.61		0.014*

Significant differences have been raised for the components of preferred food, household accidents, the age of the first sexual intercourse, social worries and depression.

*Note:*

* … t-test;

+ … Chi-Square-Test;

# … Mann-Whitney-test. Bonferroni-Correction was applied for p-values within the BBE-Questionnaire.

Most of the ICA-patients received the questionnaires by mail; four patients filled in the questionnaire in our clinic during their diagnostic routine. Return rate of the questionnaires, sent by mail, was 74%. Most participants of the control group also received the questionnaires by mail without payment; however 14 participants of this group answered the questionnaire in our clinic and received a small amount of money for participating in the study. As some of the questions asked in the questionnaire were very personal, special care was taken in telling the participants (oral or by letter) that data was handled anonymously and that there were no “good” or “bad” answers. Additionally, interviews focusing on the ability to cope with daily life were performed with those ICA-patients we saw in our daily routine.

### Materials

The questionnaire intended to obtain information about daily life functions related to olfaction. The whole questionnaire is provided as supporting online information (Questionnaire S1). For **ingestion,** participants were asked about their size, weight, preferred food and about their eating behavior. Their answers to “preferred food” were coded into food with 1 component (e.g. “pasta”, “soup”), 2 components (e.g. “rice with vegetables”) or three components (e.g. “steak with french fries and vegetables”). For “eating behavior”, participants had to agree or disagree to three statements on a four-point-scale (arms were “I totally agree” and “I do not agree at all”) and a mean was derived from these. The questions are reported in Table 1. Furthermore we originally intended to ask about ingestion as a baby, but about half of the participants stated they were uncertain about their answers to these questions, so we decided not to analyze them. However, we were able to obtain reliable data whether participants were breast fed or not. For **environmental hazards** participants were asked about household accidents. They were asked to respond to five statements and a mean score was built (see Table 2). Additionally we asked about personal hygiene by asking the participants about the frequency of showering, and about their washing behavior (see Table 2). For **Social Behavior and Communication** participants were asked to respond to three statements of social insecurity and a mean score was built (see Table 2). Additionally participants were asked about intimate relationships by asking about the present status of partnership (married, divorced, single, engaged, widowed), self-rated well-being in the partnership (on a five-point scale), the number of children they have, the age of first sexual intercourse, self-reported sexual satisfaction (on a four-point scale) and the number of different sexual partners they had during their life. Furthermore we presented them a questionnaire for personal relationships (BBE) to obtain information about attachment towards the mother and towards the partner [24], [25]. This questionnaire consists of 14 items for attachment to the mother and 14 for attachment to the partner, formulated as statements. Participants rated their agreement on a five-point-Likert-scale. Two sub-scores were formed, one for attachment security and one for dependency.

**Table 2:** Items forming the subscales of eating behavior, household accidents, social insecurity and washing behavior.

**Eating behavior**	I eat at fixed times (reverse coding).
	I eat when I'm hungry.
	I eat, when I have appetite.
**Household accidents**	I have accidently eaten spoiled food.
	Accidents in my household often happen to me.
	Occasionally it happens to me, that I scorch food.
	Sometimes I burn clothes when ironing.
	I rarely perceive smoke.
**Social insecurity**	I have problems in contacting other people.
	I worry about my body odor.
	I avoid eating with other people.
**Washing behavior**	I wash myself at fixed times (reverse coding).
	I wash myself when I feel dirty.

Al of the items are to be rated on a four-point scale (“I totally agree” to “I don't agree at al”). For subscales the average of the related items is calculated.

Finally, the Beck Depression Inventory (BDI) was presented to all participants, a widely-used, standardized and validated instrument for measuring depressive symptoms [26].

### Statistical Analysis

Data were analyzed using SPSS vs. 17 (SPSS Inc., Ill, USA). T-test was used for the comparison of both groups regarding the variables *size, weight, body-mass index, age of first sexual intercourse, number of sexual relations, components of preferred food*, as well as *BDI-questionnaire* and *BBE-questionnaire*. Bonferroni Correction was applied for p-values within the BBE-Questionnaire. For the comparison of *partnership status* and *breast feeding* Chi-Square testing was applied. All of the other variables, namely *eating behavior, household accidents, washing behavior, frequency of showering, satisfaction with partnership* and *sexual satisfaction* were analyzed using Mann-Whitney test. The level of significance was set at 0.05.

## Results

The results are reported focusing on the main olfactory functions mentioned above.

### Ingestion

For ingestion, we found no significant difference in breast feeding. For the controls 85% indicated they had been breast fed, but for the IAC-patients 78.1% had been. There was also no significant difference in the weight, size or in the Body-Mass Index of both groups. Both groups did not differ in their eating behavior (see Figure 1 and Table 1).

### Environmental Hazards

**Figure 2:**
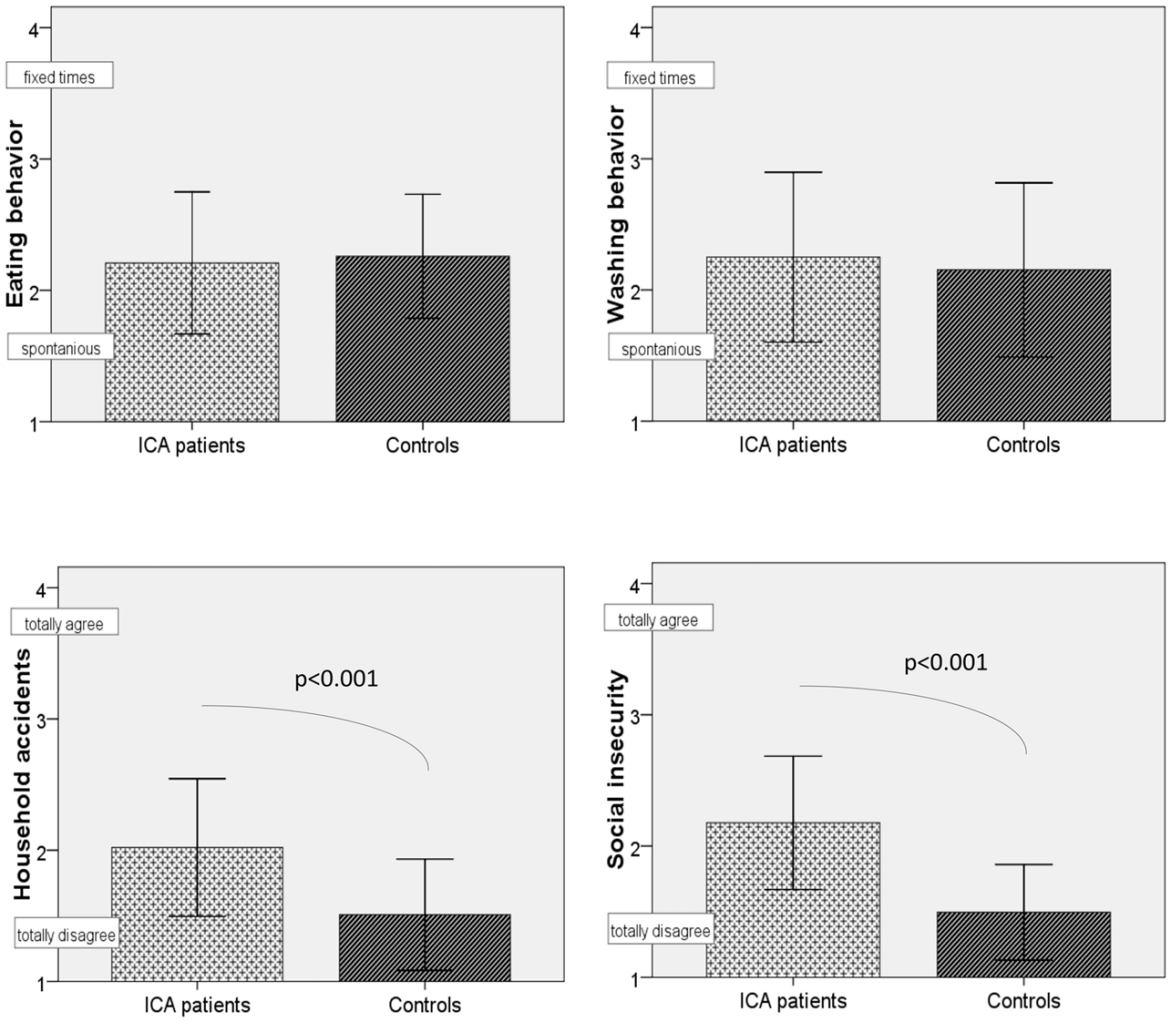
Comparison of ICA-patients (N = 32) and age-matched controls (N = 36) with regard to eating behavior, washing behavior, household accidents and social insecurity. The bars visualize the mean ratings for the scales; error bars indicate the single standard deviation. ICA-patients significantly more often agreed to have household accidents and to be unsure in certain social situations.

ICA- patients reported more household accidents than healthy controls (p = 0.001, see Table 1 and Figure 2). For personal hygiene, however, no significant difference in the frequency of showering could be revealed. There was also no significant difference in the ratings of washing behavior (see Figure 2 and Table 1).

### Social Behavior and Communication

ICA-patients and controls did not differ significantly in their rated attachment towards mother or partner in the BBE-questionnaire. Ratings of both groups were within a normal range [24], [25] (see Table 1).

ICA-patients reported more worries about social situations than controls (p<0.001), i.e. they reported worrying about their own body odor, having problems in interactions with other people and avoided eating with others (see Table 1 and Figure 2).

**Figure 3:**
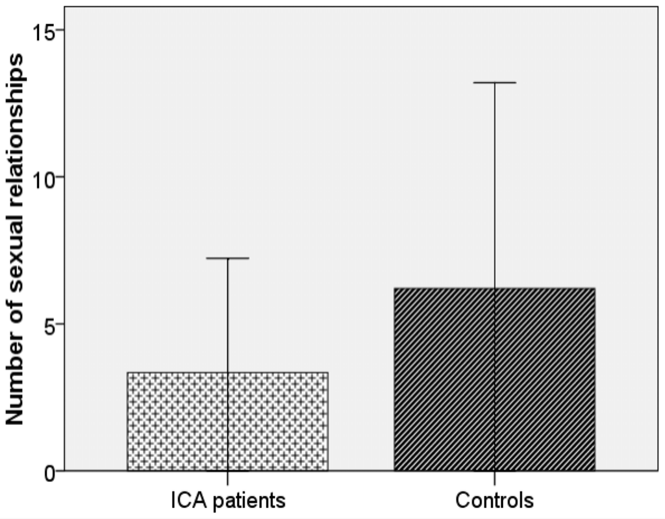
Comparison of the number of sexual relationships in ICA-patients (N = 32) and age-matched controls (N = 40). Controls report to have had significantly more different sexual partners than ICA-patients.

For partnership and sexual behavior, there was no significant difference between both groups in the partnership status or the self-reported satisfaction with their partnership. There were also no significant differences in the age of the first sexual intercourse, the self reported sexual satisfaction or in the number of children. However, ICA-patients reported to have had significantly less sexual partners than controls (p = 0.031, see Table 1 and Figure 3).

### Depression

ICA-patients exhibited higher scores in the Depression Inventory compared to controls (p = 0.018, see Table 1).

## Discussion

Based on our data, ICA-patients do not seem to differ a lot in their daily life functions from healthy controls. Does this mean that the sense of olfaction is dispensable for humans, at least in the Western world? We do not believe this to be true. Although people who were never able to smell, seem to cope well with this deficit, there are some restrictions, very worthy of further study. Likewise, the domains where we found no differences between people with and without a sense of smell are very interesting and raise further questions about the role of the sense of smell in daily life.

For ***ingestive behavior***, ICA-patients reported they were breast fed as frequently as the controls. This is very interesting because studies suggest that breast-feeding in mammals very much depends on the ability of the newborns to find the nipples by olfactory cues. In rabbits for example, the offspring has almost no chance of survival if they are not able to smell [27]. Interestingly, human mothers seem to be able to compensate for the olfactory deficit of their babies very well, perhaps because humans normally have to focus on only one baby.

As adults, ICA-patients do not differ in size or weight, have preferred foods and do not exhibit major differences in their eating behavior. As taste is a combined sensation of olfactory, tactile and gustatory senses, the lack of olfaction since birth does not seem to lead to a lack of taste.

Another function of the olfactory system is the avoidance of ***environmental hazards*** [1]. Here, ICA-patients report more household accidents. This fits information about patients who lost their sense of smell in adulthood [11]. The patients told us about different strategies to cope with this, like not leaving the iron alone or asking others whether the milk was still palatable. For hygiene behavior, on the other hand, no significant differences between both groups could be found. Nevertheless, patients reported coping strategies for hygiene, like washing clothes after a certain routine.

Not very surprisingly, for ***social behavior and communication*** there were no hints for major disturbances in relationships with others, like mother or partner. ICA-patients seem to be able to find an intimate partner and to develop a satisfying relationship as frequently as people with normal senses of smell. On average, they start their sexual behavior at the same time as age-matched controls, are as satisfied with their sexual life and have the same number of children. However, we found an ***enhanced social insecurity in ICA-patients***. This social insecurity seems to refer to persons, who are not well-known, like colleagues or distant acquaintances. As olfactory cues are able to confer social information about others [1], [2], it is possible that ICA-patients have more problems in assessing other people, because this channel of communication is closed. This may result in an increased social insecurity, which may explain the finding that ICA-patients had only about half of the number of sexual relationships of controls. Alternatively it is conceivable, that the absence of the sense of smell in sexual intercourse leads to a generally lower interest in sexual relationships. In this regard the present study does not provide satisfying answers. However, a closer look into sexual behavior in the absence of a sense of smell would be very interesting (compare [28]).

The ***enhanced ratings of depressive symptoms in ICA patients*** may also relate to the absence of the sense of smell. In fact, patients with acquired olfactory loss typically show signs of depression [9], [14] However, one could counter that those patients were affected by the loss of the sense of smell (rather than its absence), which is not the case in congenital anosmia. Another possible explanation refers to the application of the questionnaire. Some of the healthy controls received money for participating while the ICA-patients were asked to fill the questionnaires because of their deficit. However, we additionally compared both groups of healthy controls. We could find no significant difference in depression scores between those control participants who answered the questionnaire at home and those who answered it in our laboratory and received a small amount of money. This supports the hypothesis of increased risk for depression in the absence of smell.

There are several studies in humans that support a link between depression and the absence of olfaction. Reduced olfactory sensitivity has been found in patients suffering from Major Depression [29], [30], [31] and in a line with this, we recently found reduced olfactory bulb volume in depressed patients [32]. One of the hypotheses discussed about this depression-olfaction-coherence refers to shared functionality in limbic and para-limbic brain networks.

### Conclusion

ICA-patients differ only slightly in daily life functions related to olfaction. These differences are increased social insecurity, enhanced risk for depressive symptoms and enhanced risk for household accidents. In these domains the sense of olfaction seems to play a key role. Further research with focused assessment would be desirable.

## Supporting Information

Questionnare S1 Questionnaire used to obtain information about daily life functions related to olfaction.
(DOCX)
