# Supplementary material for: Learning about the Functions of the Olfactory System from People without a Sense of Smell
Source: PLoS One. 2012 Mar 21;7(3):e33365. doi: 10.1371/journal.pone.0033365 (PMC3310072; doi:10.1371/journal.pone.0033365)
Supplement: Questionnare S1 — Questionnaire used to obtain information about daily life functions related to olfaction. (DOCX) [file pone.0033365.s001.docx]

# Supplementary material – Questionnaire

# General Questions

age: _________

sex: _________

size: _________

weight: _________

status of partnership: 🞏married 🞏divorced 🞏single 🞏engaged 🞏widowed

number of children: _________

# Childhood development

**Have you been breast fed?** 🞏Yes 🞏no

**If yes, up to which month have you been breast-fed? ___________month**

How certain are you about this answer?

🞏Very sure 🞏relatively sure 🞏relatively unsure 🞏very unsure

**How tall and heavy have you been as toddler (till the age of three)?**

🞏above-average height 🞏average height 🞏below average height

🞏above-average weight 🞏average weight 🞏below average weight

How certain are you about this answer?

🞏Very sure 🞏relatively sure 🞏relatively unsure 🞏very unsure

**Until which month of life did you use diapers during the night? ___________months**

How certain are you about this answer?

🞏Very sure 🞏relatively sure 🞏relatively unsure 🞏very unsure

# Current feeling

Eating behavior

What is you preferred food? _____________________

|  | Totally agree | Rather agree | Rather disagree | Totally disagree |
| --- | --- | --- | --- | --- |
| I eat at fixed times |  |  |  |  |
| I eat when I am hungry. |  |  |  |  |
| I eat when I have appetite. |  |  |  |  |
| I have accidently eaten spoiled food. |  |  |  |  |
| I avoid eating with other people. |  |  |  |  |
| It happens to me that I scorch food. |  |  |  |  |

Daily life

|  | Totally agree | Rather agree | Rather disagree | Totally disagree |
| --- | --- | --- | --- | --- |
| Accidents in my household often happen to me. |  |  |  |  |
| I rarely perceive smoke. |  |  |  |  |
| Sometimes I burn clothes when ironing. |  |  |  |  |
| I worry about my body odor. |  |  |  |  |
| I have problems in contacting other people. |  |  |  |  |
| I wash myself at fixed times |  |  |  |  |
| I wash myself when I feel dirty. |  |  |  |  |

How often do you shower?

🞏more than ones a day 🞏daily 🞏every second day

🞏 more than ones a week 🞏weekly 🞏 less than ones a week

Partnership

At which age have you had you first sexual intercourse? ____years

How many different sexual partners did you have during your life? _________

Do you life in a partnership at the moment? 🞏yes 🞏no

If yes, how well do you feel in your partnership?

🞏 totally happy 🞏rather happy 🞏partly happy 🞏rather unhappy 🞏totally unhappy

If yes, how satisfied are you with you sexuality?

🞏 totally satisfied 🞏rather satisfied 🞏rather unsatisfied 🞏totally unsatisfied
